# Dimensional Analysis of Workpieces Machined Using Prototype Machine Tool Integrating 3D Scanning, Milling and Shaped Grinding

**DOI:** 10.3390/ma13245663

**Published:** 2020-12-11

**Authors:** Piotr Jaskólski, Krzysztof Nadolny, Krzysztof Kukiełka, Wojciech Kapłonek, Danil Yurievich Pimenov, Shubham Sharma

**Affiliations:** 1 Department of Production Engineering, Faculty of Mechanical Engineering, Koszalin University of Technology, Racławicka 15-17, 75-620 Koszalin, Poland; piotr.jaskolski@tu.koszalin.pl (P.J.); krzysztof.kukielka@tu.koszalin.pl (K.K.); wojciech.kaplonek@tu.koszalin.pl (W.K.); 2 Department of Automated Mechanical Engineering, South Ural State University, Lenin Prosp. 76, 454080 Chelyabinsk, Russia; danil_u@rambler.ru; 3 Department of Mechanical Engineering, IK Gujral Punjab Technical University, Jalandhar-Kapurthala Road, Kapurthala, Punjab 144603, India; shubham543sharma@gmail.com

**Keywords:** dimension accuracy, shape accuracy, 3D scanning, milling, shaped grinding, complete machining

## Abstract

In the literature, there are a small number of publications regarding the construction and application of machine tools that integrate several machining operations. Additionally, solutions that allow for such integration for complex operations, such as the machining of shape surfaces with complex contours, are relatively rare. The authors of this article carried out dimensional analysis of workpieces machined using a prototype Computerized Numerical Control (CNC) machine tool that integrates the possibilities of 3D scanning, milling operations in three axes, and grinding operations using abrasive discs. The general description of this machine tool with developed methodology and the most interesting results obtained during the experimental studies are given. For a comparative analysis of the influence of the machining method on the geometric accuracy of the test pieces, an Analysis of Variance (ANOVA) was carried out. The obtained results show that for four considered features (deviations of flatness, vertical parallelism, opening dimensions, and opening cylindricality), no statistically significant differences were detected. For the evaluation criteria, the probability level *p* exceeded the assumed confidence level α = 0.05 and ranged from *p* = 0.737167 to *p* = 0.076764. However, such differences were found for two others—a dimensional deviation between flat surfaces (*p* = 0.010467) and horizontal parallelism deviation (*p* = 0.0)—as well as for the quality of the machined surface defined by four surface texture parameters: *Ra* (*p* = 0.831797), *Rt* (*p =* 0.759636), *Rq* (*p* = 0.867222), and *Rz* (*p* = 0.651896). The information obtained by the ANOVA will be useful for the elimination the weaknesses of the prototype machine tool, further analysis of technological strategies, and to find possible benefits of integrating machining operations.

## 1. Introduction

There is a significant increase in the share of variable curvature surfaces, as presented by Yang et al. [1,2,3], in various types of engineering structures, such as: dies, molds, stamping dies, turbine elements, and decorative, design, and utility products. Designing such elements’ surfaces is not difficult thanks to the use of computer-aided design (CAD) and computer-aided manufacturing (CAM) systems. Such systems can be used to create complex parts, but in many cases, the surfaces of the parts after the milling process require grinding and precise smoothing to achieve specific quality requirements of the finished product. These operations are carried out on separate machine tools, which differ in their control.

During the production of parts, for which several machining operations have to be performed, there are timeouts due to retooling, positioning, and clamping of parts in several different machine systems. Moreover, each subsequent clamping reduces the dimensional and shape accuracy resulting from the change of machining base.

To attempt to solve this important problem in modern manufacturing, so-called complete machining (integrated machining) carried out, which integrates high-accuracy 3D scanning and Computerized Numerical Control (CNC) machining. The 3D scanning was carried out to obtain spatial information about the scanned object (points cloud) and generate a CAD model. Based on such a model, the control program for a CNC machine tool equipped with multiple tool heads and a single workpiece clamp was prepared. The technical solution ensuring the possibility of complete machining is a platform that connects a 3D scanner and a CNC machine tool, supported by appropriate computer software. Integrated machining systems have been successfully developed for over thirty years and are used in many fields of modern science and technology. Examples of some of them are given in Table 1.

Developing the above solutions, over time, allowed their commercialization and implementation as a new class of technological machines in the industry. Examples of such solutions include machine tools:

- DMG MORI CTX gamma 2000 TC;
- DMG MORI LASERTEC 65 3D;
- Doosan PUMA SMX3100L.

A CTX gamma 2000 TC (DMG Mori Seiki AG, Bielefeld, Germany) lathe and milling center is a universal machine tool integrating turning, milling, and grinding operations. On this machine tool, it is possible to process elements with diameters of up to 700 mm and lengths of up to 2050 mm. It is equipped with two spindles: one for turning with a maximum speed of 4000 rpm and one for milling with a maximum speed of 20,000 rpm. Additionally, the DMG MORI CTX gamma 2000 TC lathe and milling center offers the possibility of internal and external cylindrical grinding, face grinding, and also allows for the dressing of grinding wheels. Other applications were presented by Bleicher et al. [10].

Whereas the milling center LASERTEC 65 3D hybrid (DMG MORI Seiki AG, Bielefeld, Germany), a hybrid is a machine tool that integrates the possibility of carrying out milling and laser surfacing operations in five-axis, The machine has an operating range of 735 mm on the X-axis, 650 mm on the Y-axis, and 560 mm on the Z-axis. It is equipped with a spindle with a maximum speed of 20,000 rpm and a fiber-coupled laser diode of 3000 W. Both described machine tools of DMG MORI are controlled by the COLEOS^®^ system (DMG MORI Seiki AG, Bielefeld, Germany), which allowed us to control the production by monitoring and supervising machining process the workpiece. Some of the applications of the LASERTEC 65 3D hybrid were given by Seidel et al. [11], Bennett et al. [12], Kledwig et al. [13], as well as Soshi et al. [14,15].

The Doosan PUMA SMX3100L (Ellison Technologies, Inc., Santa Fe Springs, CA, USA) lathe and milling center is a machine tool that integrates turning and milling operations in one clamping. On this machine tool, it is possible to process workpieces with diameter up to 660 mm and length up to 2540 mm. It is equipped with two spindles: one for turning with maximum speed up to 3000 rpm and one for milling operation with maximum speed 12,000 rpm. The machine is programmed and controlled by the FANUC control system (FANUC Ltd, Yamanashi-ken, Japan). 

Specialized machine tools are not always required to integrate operations. There are solutions on the market that allow many types of operations to be performed on a CNC turret lathe with a driven *Y*-axis. Among the most interesting in this area are the tool holders offered by M.T. S.r.l. (San Giovanni In Marignano RN, Italy):

- X11 Powered Electrospindle;
- Broaching Toolholder;
- Driven Gear Hobber;
- Laser cutting device for turret lathes.

The X11 Powered Electrospindle by M.T. is a device mounted in one of the slots in the turret magazine of a CNC lathe. It enables grinding, engraving, and drilling operations at high speed. The motor of the device is driven by a system that converts the rotary movement of the driven tool holder into electrical energy driving the shaft. The device is connected to the cooling system in the turret of the CNC lathe, which enables cooling of the electrospindle motor. Moreover, the device has a dedicated system ensuring proper protection of the electrospindle against contamination and coolant during machining, thanks to which it can be installed in CNC lathes without compressed air connections. The X11 Powered Electrospindle by M.T. has a diameter of 42 mm and its maximum speed is 60,000 rpm.

The Broaching Tool holder from M.T. is also a tool holder mounted in one of the slots in the turret magazine of a CNC lathe. It is used to perform broaching machining, as a result of which it is possible to make keyway grooves directly on the CNC lathe. The 1:4 reduction factor (1600 rpm, corresponding to 400 tool strokes per minute) allows the machining of hard materials even with limited power available on the driven tool holder. Additionally, the tool holder has a double guide mechanism that increases stability during broaching and lowers the tool during the return stroke, maximizing tool life.

Driven Gear Hobber by M.T. is a tool holder designed to be mounted in one of the slots in the turret magazine of a CNC lathe. The holder enables machining of cylindrical gears with straight or helical teeth and bevel gears with straight teeth. The Driven Gear Hobber tool holder by M.T. is available in five variants differing in diameter and length of milling gears. It is available with a drive reduction factor of 1:1 or 2:1, enabling the machining of various types of gears even with limited power available on a driven turret head.

The laser cutting device for turret lathes by M.T. is a tool holder enabling laser cutting of workpieces mounted in the spindle of a CNC turret lathe. The power of the laser beam can be adjusted in a continuous or pulsed mode to enable the cutting of thick materials. The tool holder consists of elements such as:

- laser head;
- PLC controller for numerical control;
- a device for automatically picking up and depositing the laser head into a dedicated warehouse mounted near the CNC lathe spindle.

The described solutions show that it is possible to integrate several machining operations on one machine tool; however, these are still few examples, and there are still many solutions missing to allow such integration for more demanding operations—e.g., for machining of contoured surfaces with complex contours, as presented by Mikolajczyk et al. [16].

This article describes the results of the first stage of research on the multiaxis integrated milling and grinding performed on a specialized prototype machine tool additionally equipped with a 3D scanner. This research aimed to determine the achievable dimensional and shape accuracy of workpieces machined with the use of a prototype machine tool concerning reference parts shaped on a precision multiaxis machining center of one of the renowned manufacturers of machine tools.

## 2. Materials and Methods

### 2.1. Dedicated Test Piece

**Figure 1:**
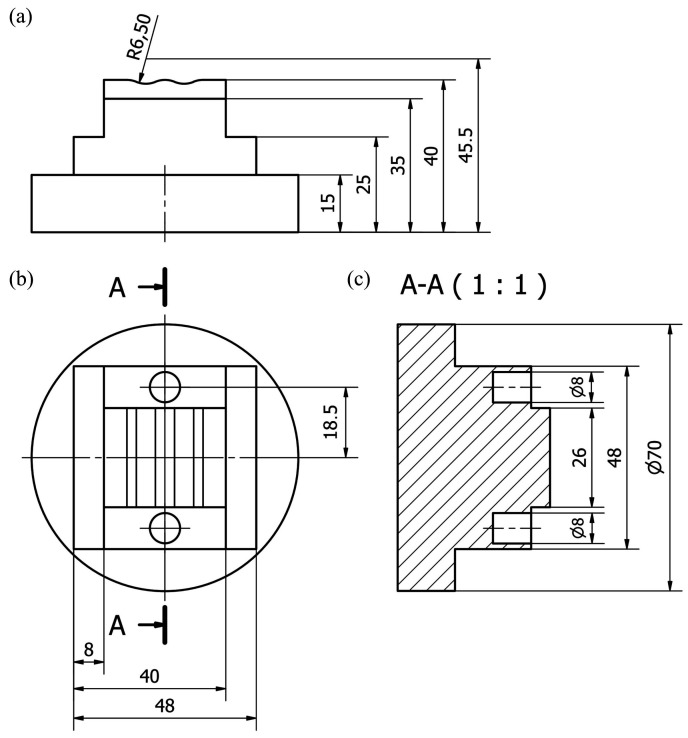
Dimensions of the test piece designed for experimental investigations of dimensional and shape accuracy: (**a**) front view; (**b**) top view; (**c**) cross-section A-A (all dimensions in mm).

The analyses of dimensional and shape accuracy of workpieces machined using a prototype machine tool integrating 3D scanning, milling, and smoothing of shaped surfaces were carried out on a specially designed dedicated element shown in Figure 1.

For the test piece, a CAM machining program has been developed for two machine tools: the VF-2 milling center (HAAS Automation, Oxnard, CA, USA) on which reference pieces were made, and the prototype machine tool integrating 3D scanning, milling, and shaped grinding.

### 2.2. Prototype Machine Tool Integrating 3D Scanning, Milling, and Shaped Grinding

**Figure 2:**
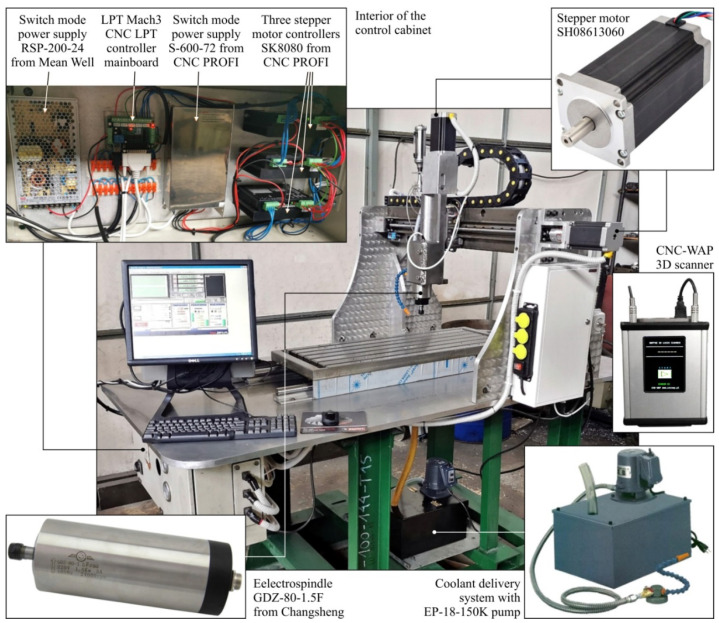
General view and key components of the prototype machine tool integrating 3D scanning, milling, and grinding of shaped surfaces.

The prototype machine tool integrating 3D scanning, milling, and shaped grinding is a numerically controlled machine tool with a built-in 3D scanner and is presented in Figure 2.

The machine tool is controlled by a desktop computer with Mach3 (Newfangled Solutions LLC, Livermore Falls, ME, USA) software installed. This program allows us to transform the PC into a CNC machine control, in which a preview of the toolpath and parameters of the machining process is displayed. The Mach3 program allows one to control the machine based on G-codes, which can be typed manually or generated in CAM programs [7,17,18]. The G-codes are designed to define the operating parameters of the spindle and stepper motors for each axis individually. For the purposes of these tests, the G-codes for each machining process were generated in Inventor CAM 2020 (Autodesk, Inc., Mill Valley, CA, USA).

The operating table of the machine tool has dimensions of 1000 × 350 mm, while the working range is 1250 mm on the X-axis, 570 mm on the Y-axis, and 180 mm on the Z-axis. The spindle moves concerning the work table in three axes and is driven by three SH08613060 stepper motors, which transfer the drive to the traction screws. The motors used have a resolution of 200 steps/rev, a current consumption per 6A coil, and a holding torque of 13 Nm. The tool drive system in the prototype machine tool consists of a GDZ-80-1.5F (Changsheng, Guangzhou, China) electrospindle and a 1.5 kW-3F (Poltech S.C., Kraków, Poland) inverter. The developed machine tool also uses a coolant delivery system with an EP-18-150K pump. It consists of a coolant tank, coolant pump, coolant discharge hose, and a coolant supply hose ended with an articulated hose to direct the outgoing coolant.

The spatial scanning subsystem is based on a 3D CNC-WAP scanner (CNC-WAP, Sanok, Poland). It is a universal device that can be used in any numerically controlled milling machine. Its operating field in the X and Y axes is compatible with the CNC machine tool’s working range, while the scanning range in the *Z*-axis is a maximum of 70 mm. The scanner operates with dedicated WAPP 3D software (CNC-WAP, Sanok, Poland), which enables:

- generation of G-codes for the CNC machine to perform the spatial scanning process;
- editing of 3D model geometry;
- saving of the scanned geometry of the model in the formats *.stl, *.dxf and *.bmp.

As a result, the described machine tool allows for spatial scanning, three-axis milling, and for grinding (smoothing) operations with the use of flexible abrasive tools (e.g., abrasive discs).

### 2.3. Machining Center for Making Elements and Verification of Dimensional and Shape Accuracy

The test pieces shown in Figure 1 were made for comparison purposes on the HAAS VF-2 milling center (HAAS Automation, Oxnard, CA, USA). This machine allows for machining in three axes with a working range of 762 mm on the X-axis, 406 mm on the Y-axis, and 508 mm on the Z-axis. The machine spindle allows working with a maximum speed of 8100 rpm. Additionally, the machine is characterized by an enlarged tool magazine concerning the base version of this model, so that there are thirty tool slots in the magazine. The examples of some applications of the HAAS VF-2 milling center are given by Sinlah et al. [19], Cai et al. [20], Limón-Molina et al. [21], as well as Ma et al. [22].

### 2.4. Workpiece Material

The test pieces were made of 70 mm diameter AlCu4MgSi aluminum shafts. This was an alloy characterized by good strength properties as well as high tensile strength and fatigue. It is suitable for welding but is moderately resistant to corrosion. In production, it is used to make structural elements of aircraft, parts for machine construction, military equipment, and components for the automotive industry. Detailed characteristics of physical properties of AlCu4MgSi aluminum are presented in Table 2.

### 2.5. Machining Parameters and CAM Machining Program

The same machining parameters were programmed for the HAAS VF-2 milling center and for the prototype machine tool integrating 3D scanning, milling, and smoothing of contoured surfaces. The designed elements were made using two Mastermet (Wrocław, Poland) tools. For machining the contours of the elements, the MM06.55.3.AL flat milling cutter (Mastermet, Wrocław, Poland) was used, which was programmed for the spindle speed of 8000 rpm and feed speed of 600 mm/min. For contouring, the ball milling cutter MM06.55.2.R3 (Mastermet, Wrocław, Poland).Al was used, which is programmed for a spindle speed of 6000 rpm and a feed rate of 240 mm/min.

For the CAM machining program, Autodesk Inventor 2020 with CAM Ultimate 2020 (Mill Valley, CA, USA) was used. The program defined the selected tools and machining parameters, defined the pig iron in the form of a cylinder with a diameter of 70 mm and a height of 41 mm, and then set the base point on the machined roller center. The next step was to select individual machining operations. For this purpose, the following operations were selected for machining:

- face planning (*face*);
- 2D roughing of the selected flat surface (*adaptive*);
- 2D finishing milling of the selected contour (*contour*);
- opening milling (*bore*);
- finishing 3D profile milling (*parallel*).

The list of technological operations for machining the test piece with assigned tools and machining parameters is presented in Table 3.

The last step in preparing a machining program was to generate a G-code in the postprocessor. G-codes were generated for two configurations:

- HAAS pre-NGC—for the HAAS VF-2 milling center;
- Mach3Mill—for a prototype machine tool integrating 3D scanning, milling, and smoothing of contoured surfaces.

**Figure 3:**
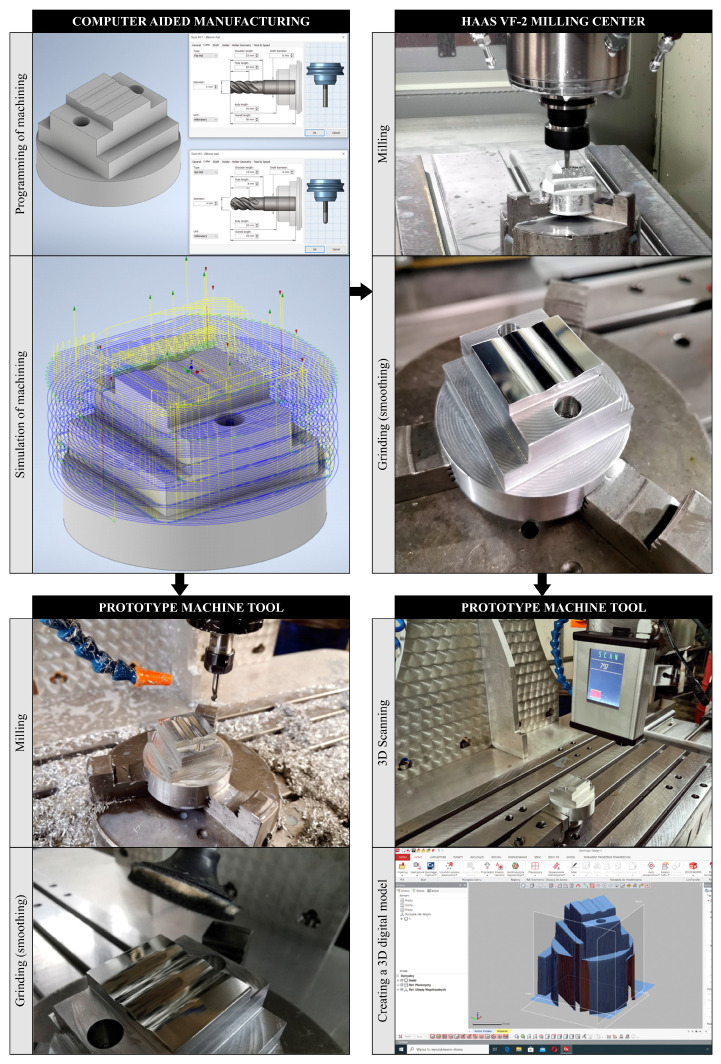
Graphical representation of the stages of experimental research methodology in the field of operations leading to the preparation of test pieces.

Three test pieces each were made on both machine tools, on which comparative analyses were carried out concerning the accuracy of dimensions, shape, and surface texture. In these analyses, each measurement was performed with the number of repetitions *n* = 3. Additionally, 3D scanning tests were carried out based on elements produced on the HAAS VF-2 machining center. Figure 3 shows, in the form of a schematic diagram, the individual stages of the experimental research methodology in the field of operations leading to a set of test elements subjected to metrological analyses.

### 2.6. Measurement Systems

**Figure 4:**
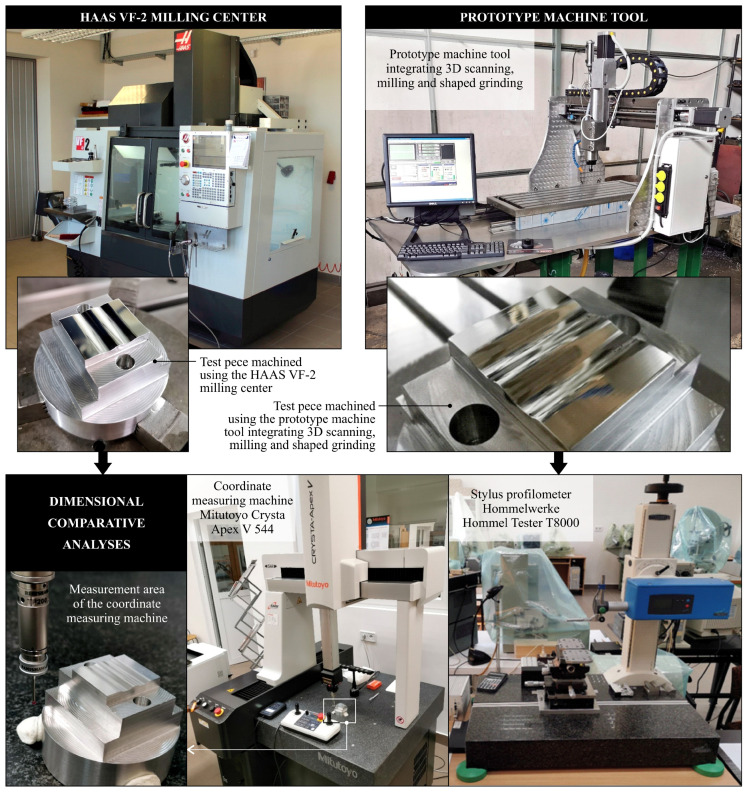
Methodology for comparative analyses of the accuracy of dimensions, shapes, and surface textures of test pieces machined using two machining machine tools.

For dimensions and surface texture analysis, two measurement systems were used. Surface texture was measured by a stylus profilometer Hommel-Tester T8000 (Hommelwerke GmbH, Villingen-Schwenningen, Germany)—Figure 4. The instrument was equipped with the following ccomponents: TKL100 pick-up (Hommelwerke GmbH, Villingen-Schwenningen, Germany) with a diamond stylus tip (opening angle: 90°, tip radius: 1.5 μm), traverse unit Waveline™ (Hommelwerke GmbH, Villingen-Schwenningen, Germany) 60 Basic (tracing length: 60 mm, resolution: 0.1 μm, tracing speed: 0.1–3 mm/s), and vertical displacement column Wavelift™ (Hommelwerke GmbH, Villingen-Schwenningen, Germany) 400M (max. traverse: 400 mm), granite plate Wavesystem™ 780 (Hommelwerke GmbH, Villingen-Schwenningen, Germany). For the control of the measurement process, the dedicated Turbo Roughness for Windows 3.1 was used, while for the analysis of measurement data and their advanced visualization, TalyMap Silver 4.1 was used using Mountains Technology™ (Digital Surf, Besançon, France).

High-accuracy dimensional analysis was carried out by a small-size portal-type multisensory coordinate measuring machine, Crysta Apex V544 (Mitutoyo Corp., Kawasaki, Japan), equipped with the PH20 5-axis touch-trigger system (Renishaw, Wotton-under-Edge, UK)—Figure 4. The instrument was characterized by: measuring range (X, Y, Z): 500 × 400 × 400 mm, measuring resolution: 0.1 µm, drive speed (max.): 519 mm/s, drive speed (during measurement): 8 mm/s, acceleration (for each axis): 1300 mm/s^2^, air supply (pressure): 0.4 MPa, dimensions: 2185 × 1082 × 1191 mm, and mass: 542 kg. The measurements and analyses carried out by the Crysta Apex V544 were supported by MCOSMOS 4.0 (Mitutoyo Corp., Kawasaki, Japan) software.

**Figure 5:**
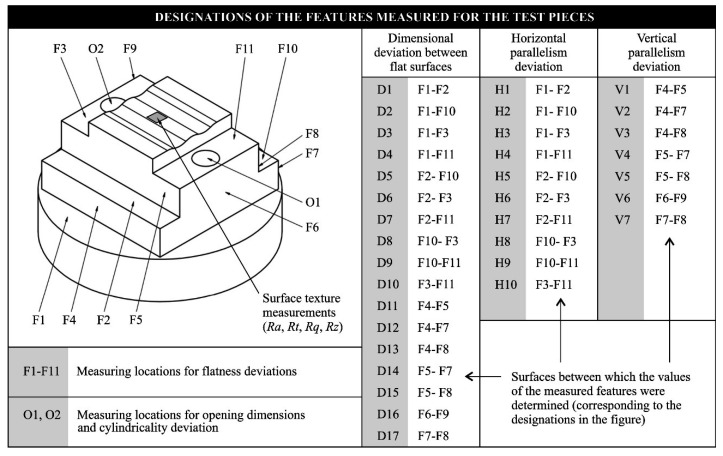
Assumed designations of the features measured for the test pieces.

Figure 4 shows a diagram presenting the methodology for comparative analyses of the accuracy of dimensions, shapes, and surface textures of test pieces machined using the HAAS VF-2 milling center (as reference) and the prototype machine tool integrating 3D scanning, milling, and shaped grinding. Figure 5 shows the assumed designations of the features measured for the test pieces.

## 3. Results and Discussion

To identify the significant effects of the input parameters and their relationship on the output parameters, one of the statistical techniques [23]—the one-way Analysis of Variance (ANOVA) presented by Tank et al. [24], Köklü et al. [25], Kumar et al. [26], as well as Palaniappan et al. [27]—was used. Statistical calculations were carried out at a confidence level of α = 0.05. If the value of the probability is *p* < α, it means that this parameter is significant. If *p* ≥ α it means that this parameter is not significant. Calculation was carried out in Statistica 13.1 (TIBCO Software Inc., Palo Alto, CA, USA) software.

**Figure 6:**
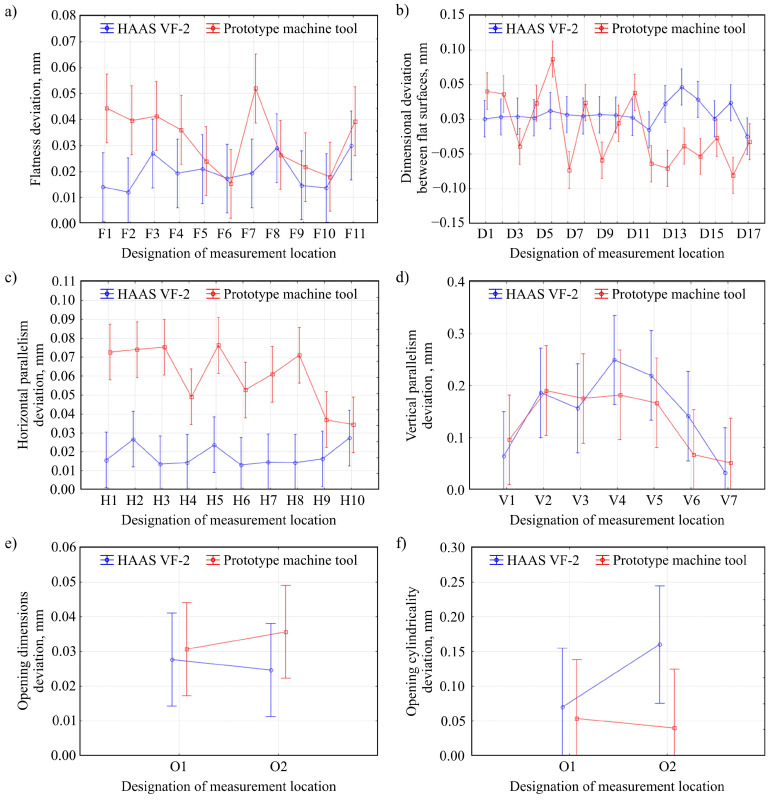
Comparison of the results of geometric measurements of test pieces machined with the use of HAAS VF-2 milling center and prototype machine tool integrating 3D scanning, milling, and shaped grinding: (**a**) flatness deviation; (**b**) dimensional deviation between flat surfaces; (**c**) horizontal parallelism deviation; (**d**) vertical parallelism deviation; (**e**) opening dimensions deviation; (**f**) opening cylindricality deviation (vertical bars present 0.95 confidence intervals, number of measurement repetitions *n* = 3).

Figure 6 shows the results of geometric measurements of test pieces machined using two analyzed machine tools (HAAS VF-2 milling center and prototype machine tool). Six parameters were selected for analysis: flatness deviation [28,29] (Figure 6a), a dimensional deviation between flat surfaces (Figure 6b), horizontal parallelism deviation (Figure 6c), vertical parallelism deviation (Figure 6d), opening dimensions deviation (Figure 6e), and opening cylindricality deviation (Figure 6f). Table 4 presents the ANOVA variance analysis of the obtained geometric measurements corresponding to the data shown in Figure 6.

The results of the analysis of the statistical significance of the influence of machining method on the geometric accuracy of the test pieces showed that in the case of four out of six evaluated features (flatness deviation, vertical parallelism deviation, opening dimensions deviation, and opening cylindricality deviation), no statistically significant differences were detected. This proves that the test pieces’ geometric accuracies were very similar for both compared machining systems: HAAS VF-2 milling center and prototype machine tool integrating 3D scanning, milling, and shaped grinding. However, the analysis of variance showed a statistically significant influence of the applied machine tool on a dimensional deviation between flat surfaces and horizontal parallelism deviation. This may be due to the insufficient rigidity of the prototype machine tool’s gantry construction. The fact that the analyses showed statistically significant differences only for the measurement results of two out of the six evaluated features suggests that only one of the prototype machine tool’s machining axes has insufficient rigidity.

**Figure 7:**
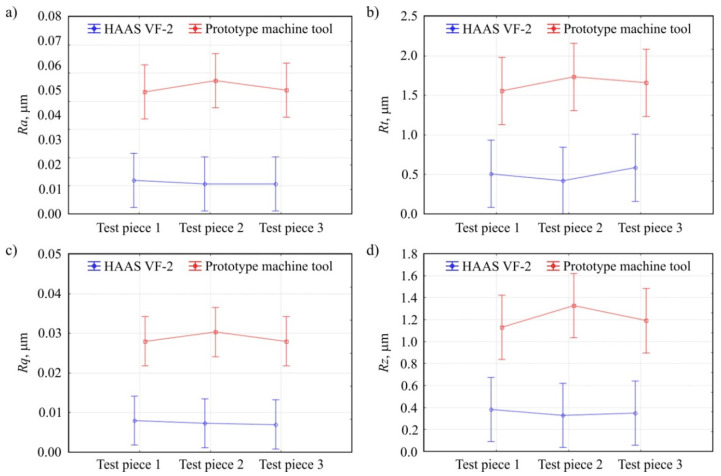
Comparison of the results of surface texture measurements of test pieces machined with the use of HAAS VF-2 milling center and prototype machine tool integrating 3D scanning, milling, and shaped grinding: (**a**) arithmetical mean deviation of the roughness profile *Ra*; (**b**) total height of the profile within a sampling length *Rt*; (**c**) root mean square deviation of the roughness profile *Rq*; (**d**) maximum height of the profile within a sampling length *Rz* (vertical bars present 0.95 confidence intervals, number of measurement repetitions *n* = 3).

Figure 7 shows the results of surface texture measurements of test pieces machined using two analyzed machine tools. Four roughness (profile) parameters were selected for analysis: arithmetical mean deviation of the roughness profile *Ra* (Figure 7a), total height of the profile within a sampling length *Rt* (Figure 7b), root mean square deviation of the roughness profile *Rq* (Figure 7c), and maximum height of the profile within a sampling length *Rz* (Figure 7d). Three data points correspond to the results of measurements of the same top surface but for three test pieces manufactured during tests using each machining tool. Table 5 presents the results of the ANOVA variance analysis of the obtained surface texture measurements corresponding to the data shown in Figure 7.

In the case of all evaluated surface texture parameters, differences can be noticed in the determined values (Figure 7). The surface roughness of the test pieces machined with the prototype machine tool was about four times higher than the surface shaped on the HAAS VF-2 milling center. This difference resulted from the course of the shape milling of the evaluated upper surface—the operation preceding the final smoothing of the surface. Despite the use of the same tools and machining parameters, the machining traces created during the milling process were not correctly smoothed during the final grinding in the case of the prototype machine tool integrating 3D scanning, milling, and shaped grinding. The assessment of the statistical significance (by ANOVA method) of the differences in the surface texture measurements obtained confirmed the significant influence of the machine tool used on the quality of the machined surface (Table 5). The probable cause of the differences shown is insufficient rigidity resulting from the type of bearings used in the kinematic system.

## 4. Conclusions

This article presents the results of measurements of dimensional and surface texture accuracy of test pieces machined using a prototype machine tool integrating 3D scanning, milling, and shaped grinding with relation to reference samples shaped on a precision multiaxis machining center by a renowned manufacturer of machines. The novelty of the presented prototype machine tool is based on the integration in one functional system of many modules that enable 3D scanning, milling, and shaped grinding. The most important aspect of such a combination of these functions is the integration in one control system, ensuring not only the control of its work but also automated creation of machining programs based on 3D scanning results. Moreover, in the future it is also planned to extend the capabilities of this machine tool with functions related to the validation of obtained machining results (also using the scanning module, analogously to the Rapid Inspection concept). Based on the analyses described, several detailed conclusions were formulated.

1. The use of Mach 3 program as a control system and CNC LPT Mach3 controller made it possible to integrate all elements of the machine tool and allowed to carry out 3D scanning, milling, and smoothing of shaped surfaces on one machine tool.
2. The shape of the developed test piece made it possible to assess both the accuracy of dimensions and shape of flat, cylindrical and shaped surfaces as well as surface texture using a multicriteria approach.
3. Results of the comparative analysis of variance by the ANOVA method of the influence of machining method on the geometric accuracy of the test pieces showed that, in four cases out of six, evaluated features (flatness deviation: *p* = 0.076764, vertical parallelism deviation: *p* = 0.737167, opening dimensions deviation: *p* = 0.510757, and opening cylindricality deviation: *p* = 0.197715) showed no statistically significant differences.
4. The same analysis showed a statistically significant influence of the applied machine tool on a dimensional deviation between flat surfaces (*p* = 0.010467) and horizontal parallelism deviation (*p* = 0.0).
5. ANOVA also indicated a statistically significant influence of the applied machine tool on the quality of the machined surface defined by four surface texture parameters: *Ra* (*p* = 0.831797), *Rt* (*p =* 0.759636), *Rq* (*p* = 0.867222), and *Rz* (*p* = 0.651896).
6. Analyses of the significance of the influence of the applied machine tool on the value of particular parameters allowed us to determine the areas of compatibility of results obtained using both compared machines.
7. The demonstrated nonconformities result from insufficient rigidity of the prototype machine tool’s gantry construction as well as from the type of bearings used in the kinematic system (in at least one of the working axes of the machine).
8. To increase the dimensional and shape accuracy of elements made with the use of the prototype machine tool, it is necessary to stiffen its structure and change the type of bearings in the kinematic system of the machine tool.
9. The carried out research allowed us to define the strengths and weaknesses of the prototype machine tool integrating 3D scanning, milling, and shaped grinding, which will lead to the elimination of the detected imperfections and further analysis of technological strategies and possible benefits of integrating machining operations.

## Figures and Tables

**Table 1:** Integrated machining systems and their fields of application.

Integrated Methods	Area of Application	Author(s)	Reference
3D scanning (triangulation) + CNC machining (milling)	Free-form surfaces and quadric surfaces	Bradley et al. (1992)	[4]
3D scanning (LDS) + NC machining	Aerospace industry, large thin-walled parts	Liu et al. (2015)	[5]
3D scanning + CNC machining (milling)	Small-engineering parts	Wu et al. (2014)	[6]
3D scanning (SLS) + CNC machining (milling)		Galantucci et al. (2015)	[7]
3D scanning (ACT) + CNC machining (milling)	Orthodontics, orthodontic denture	Chang et al. (2006)	[8]
3D scanning (triangulation) + CNC machining (milling)	Dentistry, dental restorations	Milde and Morovič (2016)	[9]

LDS—Laser Displacement Sensor, NC—Numerical Control, SLS—Slit Laser Scanner, ACT—Abrasive Computer Tomography.

**Table 2:** The physical properties of aluminum AlCu4MgSi.

Hardness	110 HB
Solidification temperature	510 °C
Pour point	645 °C
Density	2.79 g/cm^3^
Poisson number	0.33
Thermal expansion coefficient	22.9 μm/mK
Specific heat	873 J/kgK
Specific resistance	51 nWm
Conductivity	34% IACS (International Annealed Copper Standard)
Thermal conductivity	134 W/mK
Shear modulus *G*	27,200 MPa
Modulus of elasticity *E*	72,500 MPa

**Table 3:** List of technological operations for machining the test piece with assigned tools and machining parameters.

Technological Operations	Tool	Parameters	
		Feed Speed, mm/min	Spindle Speed, rpm
Face planning	MM06.55.3.AL	600	8000
2D roughing of the selected flat surface	MM06.55.3.AL	600	8000
2D finishing milling of the selected contour	MM06.55.3.AL	600	8000
Opening milling	MM06.55.3.AL	600	8000
Finishing 3D profile milling	MM06.55.2.R3.Al	240	6000
Grinding (smoothing)	Compressed nonwoven disc, 3M XL-DR, diameter 75 mm, granulation 2S FIN	50	8000

**Table 4:** Results of the analysis of variance by the Analysis of Variance (ANOVA) method of the obtained geometrical measurements (in the table, the probability values for the features under consideration are bold).

Analyzed Feature		Sum of Squares of Effects SS	Degrees of Freedom	Number of Degrees of Effects MS	*F* Test Value	Probability Level *p*	Is the Machining Method Significant?
Flatness deviation	Free term	0.045138	1	0.045138	348.1856	0.000000	Yes
	Flatness	0.003126	10	0.000313	2.4115	0.021973	Yes
	Method	0.002698	1	0.002698	20.8139	0.000040	Yes
	Flatness × Method	0.002418	10	0.000242	1.8653	**0.076764**	No
	Error	0.005704	44	0.000130	–	–	–
Dimensional deviation between flat surfaces	Free term	0.002203	1	0.002203	4.26330	0.042760	Yes
	Dimensions	0.059508	16	0.003719	7.19858	0.000000	Yes
	Method	0.016063	1	0.016063	31.08918	0.000000	Yes
	Dimensions × Method	0.073535	16	0.004596	8.89535	**0.000000**	Yes
	Error	0.035133	68	0.000517	–	–	–
Horizontal parallelism deviation	Free term	0.091963	1	0.091963	580.0884	0.000000	Yes
	Horizontal parallelism	0.003789	9	0.000421	2.6559	0.016255	Yes
	Method	0.026924	1	0.026924	169.8319	0.000000	Yes
	Horizontal parallelism × Method	0.004089	9	0.000454	2.8657	**0.010467**	Yes
	Error	0.006341	40	0.000159	–	–	–
Vertical parallelism deviation	Free term	0.837260	1	0.837260	157.6767	0.000000	Yes
	Vertical parallelism	0.156567	6	0.026095	4.9142	0.001515	Yes
	Method	0.003155	1	0.003155	0.5941	0.447293	No
	Vertical parallelism × Method	0.018725	6	0.003121	0.5877	**0.737167**	No
	Error	0.148679	28	0.005310	–	–	–
Opening dimensions deviation	Free term	0.010561	1	0.010561	104.2237	0.000007	Yes
	Opening dimensions	0.000003	1	0.000003	0.0296	0.867662	No
	Method	0.000147	1	0.000147	1.4507	0.262843	No
	Opening dimensions × Method	0.000048	1	0.000048	0.4737	**0.510757**	No
	Error	0.000811	8	0.000101	–	–	–
Opening cylindricality deviation	Free term	0.000784	1	0.000784	19.32033	0.002301	Yes
	Opening cylindricality	0.000044	1	0.000044	1.08624	0.327775	No
	Method	0.000140	1	0.000140	3.45175	0.100255	No
	Opening cylindrica-lity × Method	0.000080	1	0.000080	1.97331	**0.197715**	No
	Error	0.000325	8	0.000041	–	–	–

**Table 5:** Results of the analysis of variance by the ANOVA method of the obtained surface texture measurements (in the table, the probability values for the features under consideration are bold).

Analyzed Feature		Sum of Squares of Effects SS	Degrees of Freedom	Number of Degrees of Effects MS	*F* Test Value	Probability Level *p*	Is the Machining Method Significant?
Arithmetical mean deviation of the roughness profile *Ra*	Free term	0.352800	1	0.352800	242.3817	0.000000	Yes
	*Ra*	0.000233	2	0.000117	0.0802	0.923465	Yes
	Method	0.128356	1	0.128356	88.1832	0.000001	Yes
	*Ra* × Method	0.000544	2	0.000272	0.1870	**0.831797**	Yes
	Error	0.017467	12	0.001456	–	–	–
Total height of the profile within a sampling length *Rt*	Free term	20.90889	1	20.90889	181.7901	0.000000	Yes
	*Rt*	0.02431	2	0.01216	0.1057	0.900536	Yes
	Method	5.89389	1	5.89389	51.2438	0.000012	Yes
	*Rt* × Method	0.06471	2	0.03236	0.2813	**0.759636**	Yes
	Error	1.38020	12	0.11502	–	–	–
Root mean square deviation of the roughness profile *Rq*	Free term	0.590422	1	0.590422	243.1945	0.000000	Yes
	*Rq*	0.000544	2	0.000272	0.1121	0.894855	Yes
	Method	0.204800	1	0.204800	84.3570	0.000001	Yes
	*Rq* × Method	0.000700	2	0.000350	0.1442	**0.867222**	Yes
	Error	0.029133	12	0.002428	–	–	–
Maximum height of the profile within a sampling length *Rz*	Free term	11.09205	1	11.09205	205.4929	0.000000	Yes
	*Rz*	0.01743	2	0.00872	0.1615	0.852697	Yes
	Method	3.33681	1	3.33681	61.8181	0.000004	Yes
	*Rz* × Method	0.04788	2	0.02394	0.4435	**0.651896**	Yes
	Error	0.64773	12	0.05398	–	–	–

## Nomenclature

ACT	Abrasive Computer Tomography
ANOVA	Analysis of Variance
CAD	Computer-Aided Design
CAM	Computer-Aided Manufacturing
CNC	Computerized Numerical Control
LDS	Laser Displacement Sensor
NC	Numerical Control
SLS	Slit Laser Scanner
*n*	Number of repetitions
*p*	Probability
*Ra*	Arithmetical mean deviation of the roughness profile, μm
*Rq*	Root-mean-square deviation of the roughness profile, μm
*Rt*	Total height of the profile within a sampling length, μm
*Rz*	Maximum height of the profile within a sampling length, μm
*E*	Modulus of elasticity, MPa
*G*	Shear modulus, MPa
*α*	Confidence level
